# Nonalcoholic Fatty Liver Disease: Its Mechanisms and Complications

**DOI:** 10.1155/2013/969748

**Published:** 2013-10-02

**Authors:** Abdelfattah El Ouaamari, Kaori Minehira

**Affiliations:** ^1^Section of Islet Cell and Regenerative Medicine, Joslin Diabetes Center, One Joslin Place, Boston, MA 02215, USA; ^2^Nestlé Research Center, Vers chez les Blanc, 1000 Lausanne, Switzerland

Nonalcoholic fatty liver disease (NAFLD) is a highly prevalent disorder in which fat accumulates excessively in the liver. The disease affects between 10% to 30% of the general population and up to 90% in obese patients [1]. Fatty liver, the first step of NAFLD, is linked with insulin resistance, a well-known condition that predisposes to the development of metabolic disorders such as diabetes and obesity. Although NAFLD may be asymptomatic, the development of nonalcoholic steatohepatitis (NASH) results in hepatic fibrosis, cirrhosis, and ultimately might evolve to liver cancer. While substantial progress has been made in understanding the disease, the mechanisms governing the development of hepatic steatosis and fibrosis are still unclear, and their effects on metabolism need to be clearly understood.

NAFLD has been associated with metabolic disorders, and many confounders such as hyperlipidemia, hypertension, diabetes, and obesity make research in the field very difficult to conduct. To understand the impact of NAFLD on the development of cardiovascular disease (CVD), H. Lu et al. performed a meta-analysis on 4 cross-sectional and 2 prospective cohort studies in European and Asian populations. After adjustments for confounders (age, sex, HbA1c, plasma lipid and liver enzyme levels, and metabolic syndromes), NAFLD significantly predicted CVD suggesting the prime role of the liver steatosis on the development of metabolic syndrome. 

The association between NAFLD and metabolic diseases has also been reported in children [2]. Here, L. Pacifico et al. further investigated the association between NAFLD and thyroid function in childhood obesity. In this Italian cohort of 402 overweight/obese children (age 10–12 years old), eighty-eight children (21.9%) had TSH above the normal range (>4.0 mIU/L). High TSH was associated with many metabolic variables such as hepatic steatosis, hypertriglyceridemia, elevated total cholesterol, and insulin resistance after adjustment for age, gender, pubertal status, and BMI. Stepwise multivariate regression analysis showed a significant association between hyperthyrotropinemia and hepatic steatosis (OR = 1.96). Other covariates independently associated with hepatic steatosis were hypertriglyceridemia (OR = 2.73) and insulin resistance (OR = 2.37). This study clearly demonstrated the complexity of metabolic disorders even in children. Given the growing population in obese children, it is important to understand how metabolic comorbidities develop in children in order to prevent earlier metabolic derangements.

A review from H. Rodríguez-Hernández et al. “Obesity and Inflammation: Epidemiology, Risk Factors, and Markers of Inflammation” tries to understand the causal role of low-grade inflammation on the development of different metabolic comorbidities such as CVD, diabetes, and metabolic syndrome in obese conditions. Authors concluded that abnormal levels of proinflammatory cytokines such as C-reactive protein, tumor-necrosis factor-*α*, and interleukine-6 play a central role in obesity and are strongly associated with increased risks of metabolic syndrome. These cytokines are originated from macrophage residing at adipose tissues. Obesity therefore mediates the inflammatory circumstance via increased adipose mass and leads to many metabolic disorders, such as CVD, NAFLD, and type 2 diabetes.

Another factor secreted from adipose tissue is nonesterified fatty acids (NEFA) via lipolysis. Increased fasting NEFA has been thought to induce insulin resistance and NAFLD. However, recent study on nonoxidative fatty acid disposal (fatty acids being used for VLDL or intracellular lipids synthesis) reported no correlation with whole body insulin sensitivity in obese women [3]. Therefore, F. M. Finucane et al. tried to elucidate the association between NEFA (“fasting” versus “postprandial”) and insulin sensitivity after an oral glucose loading in the Hertfordshire Physical Activity Trial (healthy elderly males). They provided evidence that impaired postprandial NEFA suppression, but not fasting NEFA, contributes to the association between whole body insulin resistance. This paper also indicated that the impaired suppression of NEFA was directly associated with increased intrahepatic lipid in elderly. 

One paper in this special issue studied the role of inflammatory pathways on the development of hepatic fibrosis. G. Willemin et al. revisited the proportional contribution of major histocompatibility class II (MHCII) molecules to the development of liver diseases. Using a model of a complete disruption of MHCII pathway, they demonstrated that in contrast to the traditional thoughts MHCII signaling was not critical to the development of steatosis-induced inflammation or fibrosis. This conclusion was build up on experiments where animals lacking MHCII challenged with high-fat diet or carbon-tetra-chloride (CCl_4_)-induced hepatic cirrhosis did develop NASH and fibrosis, respectively, at the same extent as in littermate controls. Although SNPs on MHCII genes were reported to increase a risk of hepatic inflammation and fibrosis, this study demonstrated that MHC II pathway was not required in the development of NAFLD in mice.

Three papers in this issue investigated the influence of diet or dietary supplement on hepatic steatosis. C. Gonzalez et al. reported a clinical study in which they assessed the influence of dietary intake (carbohydrate, lipid, protein, and energy) on hepatic steatosis and fibrosis in patients with NAFLD. Authors found that energy and carbohydrate intake were positively correlated with liver steatosis but not with fibrosis. Authors suggested that even moderate reductions of dietary carbohydrate might help to reduce liver fat in NAFLD. C. F. Jin et al. studied the effect of Eucommia Ulmoides Oliver cortex extracts (EUCE), a Chinese herbal extract, in the development of NAFLD induced by CCl_4_. Mice acutely treated by CCl_4_ developed hepatic steatosis and induced endoplasmic reticulum (ER) stress and oxidative stress. Pretreatment by EUCE dose dependently reduced the development of steatosis via a normalization of ApoB secretion and a reduction in ER stress, and malondialdehyde. Authors concluded that EUCE might protect liver against CCl_4_-induced hepatic lipid accumulation, ER stress and its related ROS dysregulation. This study was conducted in the case of CCl_4_-induced hepatic steatosis; it would be interesting to investigate the effect of EUCE on high-fat high-sugar diet-induced hepatic steatosis and hepatic insulin resistance. In the review article, A. B. Ross et al. emphasize the beneficial effect of whole grains versus refined grains in the prevention and treatment of NAFLD. Whole grains have higher content in many nutrients and phytochemicals than their refined counterparts. Authors proposed several possible mechanisms to improve NAFLD via increased whole grains intake: (i) reduction of total energy intake, (ii) changes to and stimulation of gut microbiota, and (iii) specific actions of phytochemicals (e.g., vitamins, phenolic acids, and betaine). This review provides us the most current information on how whole grains could impact on NAFLD. 

This special issue is dedicated to review articles, research articles and clinical studies focused on timely topics concerning liver steatosis in the context of inflammation, and how the disease relates to other metabolic disorders and the impact of diet on the development and the prevention and/or treatment of NAFLD. Divers articles in this special issue highlight broad interactions between NAFLD and metabolic disorders and its complexity. And it is evident that additional efforts are needed to gain greater insights into the mechanisms underlying the development of liver steatosis, NASH, and cirrhosis. Gained knowledge will provide opportunities for effective medications/diets for the treatment/prevention of NAFLD and associated complications.

We hope that the information published in this special issue enriches the knowledge of our readers and scholars interested in general in the field of liver diseases and in particular in NAFLD.

*Abdelfattah El Ouaamari* *Abdelfattah El Ouaamari* *Kaori Minehira* *Kaori Minehira*
